# A New Case of dic(1;15)(p11;p11) in AML M1: Apropos of a Case and a Review of the Literature

**DOI:** 10.1155/2013/462896

**Published:** 2013-02-27

**Authors:** Deniz Gören Şahin, Beyhan Durak, Eren Gündüz, Sevilhan Artan, Olga Meltem Akay

**Affiliations:** ^1^Department of Hematology, School of Medicine, Eskisehir Osmangazi University, 26480 Eskisehir, Turkey; ^2^Department of Medical Genetics, School of Medicine, Eskisehir Osmangazi University, 26480 Eskisehir, Turkey

## Abstract

Acute myelogenous leukemia (AML) develops as the consequence of a series of genetic changes in a hematopoietic precursor cell. Specific cytogenetic abnormalities have been identified by karyotype analysis in AML. One of the rare chromosomal abnormalities is a dicentric chromosome, which is defined as an aberrant chromosome having two centromeres. In the literature, a limited number of cases have been reported with dic(1;15) in myeloid disorders, but only one case has been reported with in acute megakaryoblastic leukemia. Herein, we report a case of acute myelogenous leukemia without maturation with a dic(1;15)(p11;p11), resulting in trisomy of the long arm of chromosome 1. To date, this is the second case of dic(1;15) in acute myelogenous leukemia and the first case in acute myeloblastic leukemia without maturation.

## 1. Introduction

Acute myelogenous leukemia is a progressive malignant disease, which is the result of a sequence of somatic mutations in a multipotent primitive hematopoietic cell [1]. Several risk factors and structural chromosomal abnormalities like deletions, duplications, and translocations have been identified, but the specific cause is still not clear. A rare type of translocation, which is called dicentric chromosome (dic), results from the abnormal fusion of two chromosome pieces, each of which includes a centromere with alpha satellite DNA from both chromosomes. There are a limited number of cases in the literature showing dic(1;15) in myeloid disorders [2]. To the best of our knowledge, herein we report the second case of dicentric chromosome involving chromosomes 1 and 15 with the karyotype determined as 47,XX,+1,dic(1;15)(p11;p11),+8 in acute myelogenous leukemia. Also this is the first case of dic(1;15) in acute myeloblastic leukemia without maturation.

## 2. Case Report

**Figure 1:**
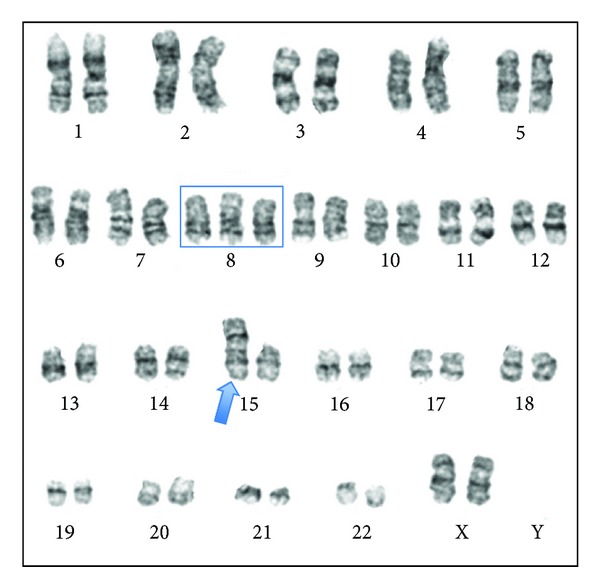
Conventional cytogenetic analysis of bone marrow aspirate sample showed the presence of 47,XX,+1,dic(1;15)(p11;p11),+8.

**Figure 2:**
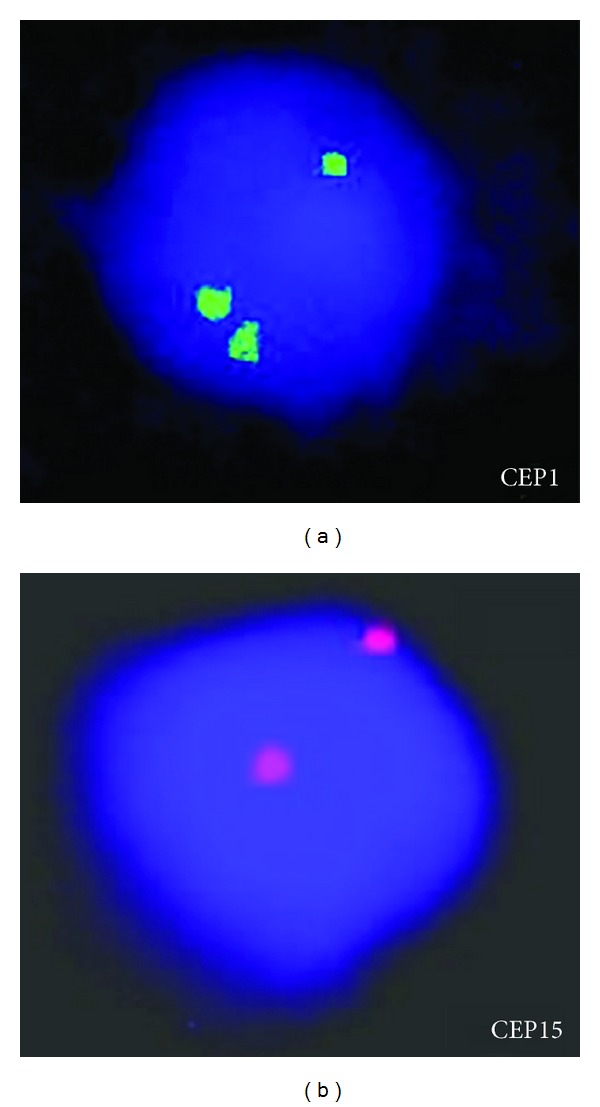
(a) Trisignal for chromosome 1 centromere. (b) Two signals for chromosome 15 centromere.

A 56-year-old woman admitted to our hospital with fatigue and tinnitus. The full blood count showed a pancytopenia with a hemoglobin level of 8.3 g/dL, a white blood cell count of 1.1 × 10^3^/*μ*L, and a platelet count of 37×10^3^/*μ*L. There was no significant abnormality in the biochemical values except slightly elevated lactate dehydrogenase level of 714 U/L. Coagulation tests were normal. Peripheral blood smear was compatible with the blood count. A bone marrow examination showed 75% of myeloblastic infiltration. Flow cytometric analyses revealed 93.8% CD34 positivity, 82.8% CD33 positivity, 90.7% HLA-DR positivity, and 30.7% myeloperoxidase positivity in the blastic cell population. CD13, CD14, CD56, and CD64 were negative. A diagnosis of acute myeloblastic leukemia without maturation was made, and remission induction chemotherapy with the 7 + 3 regimen, cytosine arabinoside (200 mg/m^2^ in a 24-hour infusion for 7 days) and idarubicin (12 mg/m^2^ intravenously for 3 days) was started. Conventional cytogenetic analysis of bone marrow aspirate sample showed the presence of 47,XX,+1,dic(1;15)(p11;p11),+8[20]/46,XX[4] (Figure 1). Fluorescent in situ hybridization for centromere specific probes showed a centromere with alpha satellite DNA from both chromosomes (Figure 2). t(8;21) was considered negative because there was no fusion signal with LSI AML1/ETO Dual Color Dual Fusion Translocation Probe. On the other hand, three ETO (8q21.3) signals confirmed trisomy 8 that was found with cytogenetic analyses. A repeat bone marrow aspiration at 4 weeks after the initiation of induction therapy revealed partial remission. She was then hospitalized for high dose cytosine arabinoside (3 g/m^2^ every 12 hour for 5 days) chemotherapy; however, the patient died of sepsis and multiple organ failure on the 21st day of hospitalization.

## 3. Discussion

Acute myelogenous leukemia (AML) results from a series of somatic mutations and/or chromosomal translocations in the majority of cases [3, 4]. Deletions of all parts of a chromosome (e.g., chromosome 5, 7, or 9) and additional chromosomes (such as trisomy 4, 8, or 13) are also common cytogenetic abnormalities in AML [5]. Besides these common cytogenetic abnormalities, there are some rare variants of translocations such as dicentric chromosomes [2]. In human leukemia, recurrent and clonal dicentric chromosomes have been found to be associated with some subtypes of leukemia, such as dic(5;17) and dic(17;18) in myeloid cell proliferations, and dic(7;9), dic(9;12) and dic(9;20) in acute lymphoblastic leukemias [6]. We report a case of acute myeloblastic leukemia with a dic(1;15)(p11;p11), resulting in trisomy of the long arm of chromosome 1. Trisomy 8 was an additional accompanied chromosomal abnormality in our case. A review of the literature showed only one case with dic(1;15)(p11;p11) in acute myeloid leukemia [7]. Dastugue et al. [7] reported a 25-year-old male who was diagnosed with acute megakaryoblastic leukemia (AML M7). He had had dic(1;15) and several cytogenetic abnormalities including trisomy 8, like in our case. In their study, the authors hypothesized that a number of cases of AML M7 may have arisen from a previously undiagnosed myelodysplastic phase. This theory was supported by the high incidence of complex karyotypes, unbalanced changes, and some other chromosomal abnormalities including dic(1;15) in their study population. There are 9 remaining cases with dic(1;15)(p11;p11) published in the literature [8–13]. Three out of these 9 cases were myeloproliferative diseases (MPD) (polycythemia vera (PV) in all 3 cases) [12, 13] and 6 out of 9 cases were myelodysplastic syndromes (MDS) (mainly refractory anemia (RA): 5 cases; RARS in one case) [8–11]. 

To the best of our knowledge, this is the first case with dicentric chromosome involving chromosomes 1 and 15 with the karyotype determined as 47,XX,+1,dic(1;15)(p11;p11),+8 in acute myeloblastic leukemia without maturation in the literature. Genes involved in dic(1;15) are unknown. However, the translocation breakpoints are likely to be in heterochromatic regions. Heterochromatin could have roles in centromere architecture and the prevention of merotely, but the possible consequences of heterochromatin rearrangements are still a question mark [6]. Heterochromatin rearrangements (partial trisomies and monosomies) cause imbalance by silencing genes placed in the region of chromosomal breakpoints due to the translocation of heterochromatin to ectopic location [14]. They also instigate functional changes of genes and proteins coupled with heterochromatin. Therefore, further studies are needed to evaluate the role of heterochromatin rearrangements in leukemia etiopathogenesis. 

Considering additional cytogenetic abnormality trisomy 8 in our case, we could speculate that one of the pathogenetically important consequences might be gene dosage. The leukemogenic potential of dic(1;15) may well be ascribed to altered gene dosages resulting from trisomy 1q and/or trisomy 8 and well-known chromosomal abnormalities found in AML (+8) as well as many solid cancers (+1q), although the critical gene targets for these chromosomal changes remain to be unveiled. 

In conclusion, this is the first case of dic(1;15) in acute myeloblastic leukemia without maturation. Molecular studies could help elucidate the pathogenetic role of this dicentric chromosome in leukemia.
